# Assessing the Utilization of Large Language Models in Medical Education: Insights From Undergraduate Medical Students

**DOI:** 10.7759/cureus.47468

**Published:** 2023-10-22

**Authors:** Sairavi Kiran Biri, Subir Kumar, Muralidhar Panigrahi, Shaikat Mondal, Joshil Kumar Behera, Himel Mondal

**Affiliations:** 1 Biochemistry, Phulo Jhano Medical College, Dumka, IND; 2 Pharmacology, Phulo Jhano Medical College, Dumka, IND; 3 Pharmacology, Bhima Bhoi Medical College and Hospital, Balangir, IND; 4 Physiology, Raiganj Government Medical College & Hospital, Raiganj, IND; 5 Physiology, Nagaland Institute of Medical Sciences and Research, Kohima, IND; 6 Physiology, All India Institute of Medical Sciences, Deoghar, IND

**Keywords:** medical students, practice, attitude, knowledge, chatgpt, intelligence, surveys and questionnaires, search engine, artificial intelligence

## Abstract

Background

Artificial intelligence (AI) has the potential to be integrated into medical education. Among AI-based technology, large language models (LLMs) such as ChatGPT, Google Bard, Microsoft Bing, and Perplexity have emerged as powerful tools with capabilities in natural language processing. With this background, this study investigates the knowledge, attitude, and practice of undergraduate medical students regarding the utilization of LLMs in medical education in a medical college in Jharkhand, India.

Methods

A cross-sectional online survey was sent to 370 undergraduate medical students on Google Forms. The questionnaire comprised the following three domains: knowledge, attitude, and practice, each containing six questions. Cronbach’s alphas for knowledge, attitude, and practice domains were 0.703, 0.707, and 0.809, respectively. Intraclass correlation coefficients for knowledge, attitude, and practice domains were 0.82, 0.87, and 0.78, respectively. The average scores in the three domains were compared using ANOVA.

Results

A total of 172 students participated in the study (response rate: 46.49%). The majority of the students (45.93%) rarely used the LLMs for their teaching-learning purposes (chi-square (3) = 41.44, p < 0.0001). The overall score of knowledge (3.21±0.55), attitude (3.47±0.54), and practice (3.26±0.61) were statistically significantly different (ANOVA F (2, 513) = 10.2, p < 0.0001), with the highest score in attitude and lowest in knowledge.

Conclusion

While there is a generally positive attitude toward the incorporation of LLMs in medical education, concerns about overreliance and potential inaccuracies are evident. LLMs offer the potential to enhance learning resources and provide accessible education, but their integration requires further planning. Further studies are required to explore the long-term impact of LLMs in diverse educational contexts.

## Introduction

The integration of technology into medical education is in progress, with artificial intelligence (AI) and large language models (LLMs) being the new addition. In an era characterized by the exponential growth of medical knowledge, the ability to efficiently access, interpret, and apply information is paramount for aspiring healthcare professionals [1]. LLMs, such as ChatGPT, Google Bard, Microsoft Bing, and Perplexity, have emerged as powerful AI tools with the potential to reshape the landscape of medical education by providing innovative solutions to the challenges posed by information overload [2].

Undergraduate medical education represents a critical phase in the development of future healthcare practitioners. It is during this formative period that students acquire the foundational knowledge, skills, and attitudes that will shape their careers. Although teacher-led education is an important part of the medical curriculum, student-led learning methods are gaining popularity due to their flexibility and better engagement [3]. Among those, self-directed learning is an integral part of competency-based medical education. LLMs can have a significant impact on self-directed or student-led learning due to their transformative potential in empowering learners to take control of their educational journey [4]. In an era where information is abundantly available but often overwhelming, LLMs provide students with a versatile tool to navigate and synthesize knowledge independently. However, the successful integration of LLMs into medical education necessitates a comprehensive understanding of how these technologies are perceived and utilized by the very individuals they are designed to serve - undergraduate medical students [5].

With this background, our research question was - "Do students recognize the potential benefits of these technologies, and to what extent do they incorporate them into their educational journey? To get the answer, we designed an online survey to explore the knowledge, attitude, and practice of LLMs in the context of medical education, as perceived and experienced by undergraduate medical students. By gaining insights into the extent of students' awareness, their attitudes toward LLMs, and their actual engagement with these AI-driven tools, this study aims to shed light on the evolving dynamics of medical education in the digital age.

## Materials and methods

Type and settings

This cross-sectional study was conducted among undergraduate medical students studying in undergraduate medical courses (MBBS) at Phulo Jhano Medical College, Dumka, Jharkhand, India, after getting approval from the Institutional Review Board (approval number 51/BIO/2023). The study was carried out by ethical guidelines, and all participants provided informed consent for participation. The research took place in an online setting, where participants completed a questionnaire hosted on Google Forms.

Development of questionnaire

To assess the knowledge, attitude, and practice of undergraduate medical students regarding the utilization of LLMs in medical education, a structured questionnaire was developed. The questionnaire consisted of the following three domains: knowledge, attitude, and practice, each comprising six questions. The development of the questionnaire involved the following steps.

First, we conducted a comprehensive review of existing literature on LLMs in medical education to identify relevant themes and constructs. Next, the questionnaire was developed with input from subject matter experts in medical education and AI technology to ensure content validity. A cognitive interview was conducted with a group of 10 undergraduate medical students to assess the clarity, comprehensibility, and relevance of the questionnaire. Feedback from pilot participants was used to refine the questionnaire further. The final questionnaire was designed to assess participants' knowledge about LLMs, their attitudes toward the technology, and their practical experiences using LLMs in the context of medical education. This questionnaire was then distributed among 30 students, and the response was obtained with a gap of seven days. The first response was used to calculate Cronbach’s alpha, and two responses were used to check the test-retest reliability using intraclass correlation coefficients (ICCs) [6,7].

The responses were coded for quantitative analysis (strongly agree = 5, agree = 4, neutral = 3, disagree = 2, strongly disagree = 1). Cronbach’s alphas for knowledge, attitude, and practice domains were 0.703, 0.707, and 0.809, respectively. For a student, the average score of a domain was calculated by adding the score of six responses and dividing the value by 6. These scores of test and retest were compared using ICCs. Obtained ICCs for knowledge, attitude, and practice domains were 0.82, 0.87, and 0.78, respectively.

Participants

We recruited undergraduate medical students for this study. Hence, any undergraduate student studying modern medicine (Bachelor of Medicine, Bachelor of Surgery) was the target participant. As the survey link was shared online with all the students, those who did not participate voluntarily were automatically excluded from the study.

Data collection methods

Data collection was carried out using the finalized questionnaire, which was distributed to undergraduate medical students through an online platform (Google Forms). Participants were contacted via email, and a link to the questionnaire was provided along with a brief explanation of the research objectives and the voluntary nature of their participation. Participants were given ample time to complete the questionnaire, and reminders were sent as needed to enhance response rates.

Data analysis

Data obtained from the completed questionnaires were subjected to a structured data analysis process. There was no missing data as all questions were made compulsory in Google Forms. Next, descriptive statistics, such as frequencies and percentages, were computed to summarize the responses to individual questionnaire items within each domain (knowledge, attitude, and practice). The responses were coded for quantitative analysis (strongly agree = 5, agree = 4, neutral = 3, disagree = 2, strongly disagree = 1). For a student, the average score of a domain was calculated by adding the score of six responses and dividing the value by 6. The chi-square test was used to compare categorical variables with expected equal division in all categories, and a statistical significance indicates that the occurrence was not by chance. The ANOVA with post hoc analysis was used to compare the mean score of knowledge, attitude, and practice. Data analysis was conducted using GraphPad Prism 9.5.0 (GraphPad Software, Boston, MA), and a p-value of less than 0.05 was considered statistically significant. The results of the data analysis are reported in the subsequent sections of this study.

## Results

A total of 172 students (95 females, 75 males, and 2 preferred not to say) participated in the study. The questionnaire was distributed among 370 students. Hence, the response rate was 46.49%. A total of 57 (33.14%) first-year, 40 (23.26%) second-year, 38 (22.09%) third-year, and 37 (21.51%) fourth-year students participated in the survey. The students were similarly distributed among years of study (chi-square (3) = 6.19, p = 0.1).

**Figure 1:**
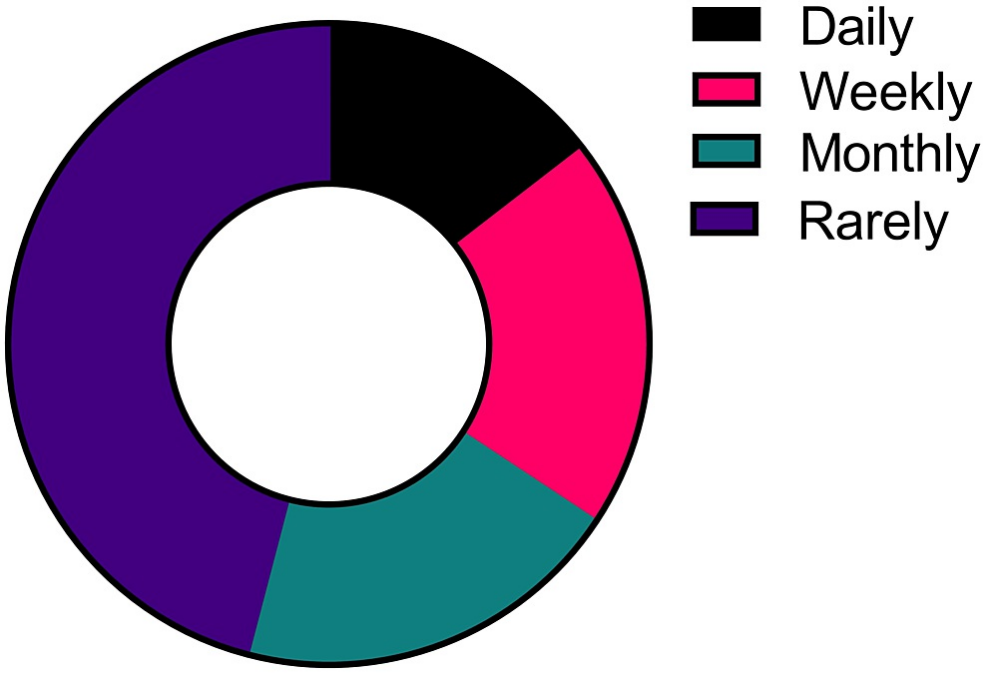
Frequency of the use of large language models by undergraduate medical students for educational purposes

The majority of the students (45.93%) rarely use the LLMs for their teaching-learning purposes (chi-square (3) = 41.44, p < 0.0001) (Figure 1).

The overall score of knowledge (3.21±0.55), attitude (3.47±0.54), and practice (3.26±0.61) were statistically significantly different (ANOVA F (2, 513) = 10.2, p < 0.0001), with the highest score in attitude and lowest in knowledge. In the post hoc test, two pairs showed significant differences - knowledge vs attitude (mean difference = -0.2607, 95% CI = -0.4042 to -0.1171, p < 0.0001) and attitude vs practice (mean difference = 0.2083, 95% CI = 0.06482 to 0.3518, p = 0.002 (Table 1).

**Table 1:** Question-wise score and average score in knowledge, attitude, and practice of using large language models for educational purposes Q, Question or statement in the questionnaire (the number after Q indicates the number of the question or statement) The data in the table are presented as mean ± standard deviation. The average score is calculated by adding the raw score of six questions and dividing the value by 6.

Domain	Q1	Q2	Q3	Q4	Q5	Q6	Average
Knowledge	3.09±1.02	2.01±1.03	3.1±0.99	2.54±0.95	3.63±0.85	3.87±0.88	3.21±0.55
Attitude	3.72±0.86	3.16±0.85	3.6±0.86	3.69±0.76	3.52±1	3.12±0.88	3.47±0.54
Practice	3±0.94	3.31±0.78	3.45±0.81	3.45±0.83	3.33±0.83	3.02±0.85	3.26±0.61

A total of 39.53% of students had an awareness of LLMs. Moreover, 61% of students believed that they understand how LLMs generate information and responses, indicating a decent grasp of their functioning. Notably, a majority (53.49%) acknowledge the potential for LLMs to be used by both teachers and students. A total of 62.79% of students find the LLMs to simplify the topic they learn. However, they are cautious about their limitations and potential for inaccuracies, with 34.88% agreeing that LLMs can generate wrong information (Table 2).

**Table 2:** Knowledge of medical students on LLMs in medical education The data in the table are presented as numbers (percentage). The p-value is of the chi-square test where a significant p-value indicates that the distribution of the responses was not by chance. LLM, large language model

Question	Strongly agree	Agree	Neutral	Disagree	Strongly disagree	p-Value
I am familiar with LLMs like ChatGPT, Google Bard, Microsoft Bing, or Perplexity	8 (4.65)	60 (34.88)	57 (33.14)	34 (19.77)	13 (7.56)	<0.0001
I understand how LLMs generate information and responses	8 (4.65)	53 (30.81)	57 (33.14)	40 (23.26)	14 (8.14)	<0.0001
LLMs can generate wrong information	12 (6.98)	48 (27.91)	68 (39.53)	34 (19.77)	10 (5.81)	<0.0001
LLM can be used both by teachers and students	18 (10.47)	74 (43.02)	55 (31.98)	19 (11.05)	6 (3.49)	<0.0001
Using LLMs helps me simplify complicated medical concepts	18 (10.47)	90 (52.33)	51 (29.65)	8 (4.65)	5 (2.91)	<0.0001
LLMs can help along with traditional learning materials like textbooks, notes, e-books, etc.	36 (20.93)	93 (54.07)	33 (19.19)	5 (2.91)	5 (2.91)	<0.0001

Medical students generally exhibit openness toward incorporating LLMs as supplementary learning tools in medical education (67.44%), recognizing the potential for comprehensive medical information and a change in how medical knowledge is accessed (31.4%). However, they also express concerns about overreliance on LLMs, which could potentially hinder the development of clinical reasoning skills (53.49%) and the risk of learning incorrect concepts (33.14%) (Table 3).

**Table 3:** Attitude of medical students on LLMs in medical education The data in the table are presented as numbers (percentage). The p-value is of the chi-square test where a significant p-value indicates that the distribution of the responses was not by chance. LLM, large language model

Question	Strongly agree	Agree	Neutral	Disagree	Strongly disagree	p-Value
I am open to including LLMs as extra learning tools for medical studies	25 (14.53)	91 (52.91)	43 (25)	9 (5.23)	4 (2.33)	<0.0001
It would help by providing comprehensive medical information	8 (4.65)	46 (26.74)	90 (52.33)	21 (12.21)	7 (4.07)	<0.0001
Medical colleges should promote the use of LLMs in the teaching-learning process	17 (9.88)	88 (51.16)	54 (31.4)	7 (4.07)	6 (3.49)	<0.0001
LLMs could change how we learn and access medical knowledge	13 (7.56)	107 (62.21)	42 (24.42)	6 (3.49)	4 (2.33)	<0.0001
Relying too much on LLMs might not develop my clinical reasoning skills	27 (15.7)	65 (37.79)	57 (3.14)	16 (9.3)	7 (4.07)	<0.0001
There is a risk of learning the wrong concept; hence, I would not blindly believe it	7 (4.07)	50 (29.07)	79 (45.93)	29 (16.86)	7 (4.07)	

Some of the medical students are actively using LLMs as supplementary resources in their medical education (30.23%). They turn to LLMs for explanations that are not readily available in traditional books (55.81%) or search engines (47.67%). Additionally, LLMs are influencing their self-study practices and increasing their confidence in discussing medical topics (37.21%) (Table 4).

**Table 4:** Practice of medical students on large LLMs in medical education The data in the table are presented as numbers (percentage). The p-value is of the chi-square test where a significant p-value indicates that the distribution of the responses was not by chance. LLM, large language model

Question	Strongly agree	Agree	Neutral	Disagree	Strongly disagree	p-Value
I use LLMs to get clearer explanations of medical topics I'm learning	5 (2.91)	47 (27.33)	76 (44.19)	31 (18.02)	13 (7.56)	<0.0001
LLMs have shown me new resources and references for my medical studies	10 (5.81)	77 (44.77)	72 (41.86)	7 (4.07)	6 (3.49)	<0.0001
I use those only when I cannot get the information in a book	6 (3.49)	90 (52.33)	58 (33.72)	11 (6.4)	7 (4.07)	<0.0001
I use those only when I cannot get the information in Google or other search engine	5 (2.91)	77 (44.77)	65 (37.79)	20 (11.63)	5 (2.91)	<0.0001
I adapt my self-study based on insights I get from LLMs	2 (1.16)	47 (27.33)	86 (50)	26 (15.12)	11 (6.4)	<0.0001
Using LLMs has made me more confident in talking about medical subjects	4 (2.33)	60 (34.88)	83 (48.26)	18 (10.47)	7 (4.07)	<0.0001

## Discussion

The finding that the majority of students rarely use LLMs for their teaching and learning purposes can be attributed to several factors. One significant factor is limited familiarity among students regarding the capabilities and benefits of LLMs in the context of medical education. Additionally, the preference for traditional learning resources, concerns about accuracy, limited integration into the curriculum, and potential barriers such as limited access or time constraints may contribute to this trend. Individual learning styles and the perceived learning curve associated with LLMs also play a role [8]. Moreover, resistance to change and the lack of training and guidance can further discourage students from incorporating LLMs into their study routines [9]. To encourage greater utilization of LLMs, the educators and institutions should address these factors by providing education on the advantages and effective use of LLMs, making them more accessible, and integrating them into the curriculum where appropriate.

The finding that knowledge scores were the lowest among students, followed by practice scores and then attitude scores, can be attributed to several factors. It suggests that while students may have a positive attitude toward LLMs in medical education and may even try to incorporate them into their practices, their knowledge about how to effectively use these models might be lacking [10]. The lower knowledge scores may indicate a need for more comprehensive training and education on the practical application of LLMs in their medical studies. Additionally, the statistical significance in the differences between knowledge vs attitude and attitude vs practice scores underscores the importance of bridging the gap between students' positive attitudes and their actual utilization of LLMs in their learning processes through targeted educational interventions and support.

The findings reveal varying levels of awareness, understanding, and perception among students regarding LLMs in medical education. A significant proportion of students are aware of LLMs and believe that they understand how they work, indicating some theoretical knowledge. They are generally positive about LLMs' potential for use by both teachers and students, as well as their ability to simplify complex topics. [11]. However, students also express caution about the potential for inaccuracies and wrong information generated by LLMs, highlighting the importance of critical evaluation. These findings suggest a need for further education and practical training on LLMs' applications to bridge the gap between awareness and effective utilization [12].

While a significant majority of students are open to incorporating LLMs as supplementary tools, this openness is tempered by concerns about overreliance on these models. Students recognize the potential for LLMs to provide comprehensive medical information and transform how they access knowledge, but they are cautious. Their apprehensions revolve around the potential negative impact on the development of critical clinical reasoning skills and the risk of acquiring incorrect concepts. This complex attitude reflects the need for a balanced approach to LLM integration, acknowledging both the benefits and potential pitfalls associated with these tools in medical education [13].

Some medical students are actively embracing LLMs as valuable supplementary resources, recognizing their capacity to provide explanations beyond what traditional books or search engines can offer. This indicates a growing recognition of LLMs as tools that can bridge gaps in understanding and enhance their learning experience [14]. Moreover, the influence of LLMs on students' self-study practices and their increased confidence in discussing medical topics reflects the adaptability of students to modern technological advancements, which empower them to take charge of their education and knowledge dissemination.

**Figure 2:**
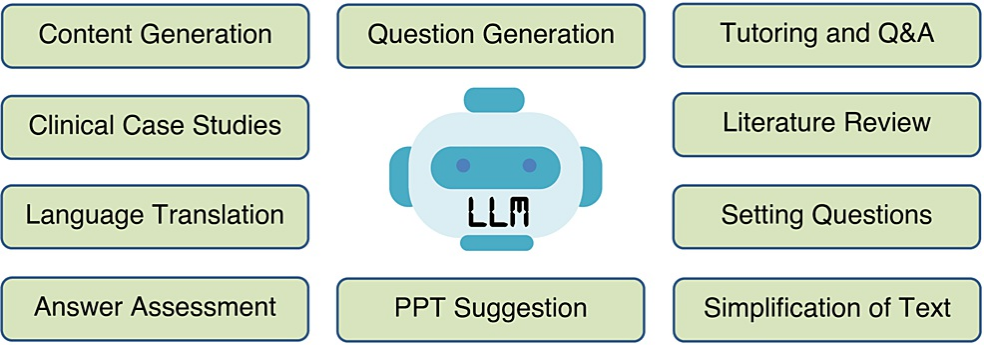
Domains of medical education where LLMs can help humans Q&A, questions and answers; LLM, large language model; PPT, PowerPoint presentation

The study's implications for medical education in developing countries are significant and offer promising opportunities for enhancing learning, accessibility, and the overall quality of medical education [15]. LLMs emerge as valuable supplementary resources, particularly in regions where access to up-to-date medical textbooks and academic materials may be limited due to resource constraints. A study by Tung and Dong found that Malayasian medical students have an awareness of AI and that they are willing to learn more about it [16]. A study by Buabbas et al. also reported a positive attitude of students toward AI in medical education. Most of the students opined that AI can help in their teaching and learning [17]. Additionally, LLMs can address faculty shortages, support research, and innovation, and foster critical thinking skills. [18]. To fully realize these benefits, educators and institutions in developing countries need to invest in faculty development and carefully integrate LLMs into their curricula, thereby harnessing the advantages while mitigating associated risks in medical education. Figure 2 summarizes the domains where LLMs can be used in medical education [19,20].

A key limitation of this study is its reliance on self-reported data from medical students, which may introduce response bias. Additionally, the study's findings are context-specific and may not be fully generalizable to diverse medical education settings, particularly those in developing countries with varying access to technology and resources. The response rate was low in the survey. Furthermore, the study does not explore the long-term impact of LLM integration into medical education or assess the effectiveness of specific educational interventions. Future research should consider these limitations and incorporate more diverse and objective measures to provide a comprehensive understanding of LLMs' role in medical education.

## Conclusions

The study reveals the current knowledge, attitude, and practice of using LLMs in medical education in an Indian medical college. While there is a generally positive attitude toward their incorporation, concerns about overreliance and potential inaccuracies are evident. LLMs offer the potential to enhance learning resources and provide accessible education, but their integration requires careful planning, faculty development, and the cultivation of critical thinking skills. This research underscores the evolving role of technology in medical education and calls for further studies to explore the long-term impact of LLMs in diverse educational contexts.

## Appendices

The questionnaire used in this study can be used for any non-commercial research or academic purposes without any permission. The questionnaire can be created from the questions or statements in Tables 2, 3, and 4. A pdf of the questionnaire with consent and instruction can be obtained from Dr. Himel Mondal, Assistant Professor of Physiology, All India Institute of Medical Sciences, Deoghar, Jharkhand, India, via email ().
